# Retraction Note: Long non-coding RNA BRE-AS1 inhibits the proliferation, migration, and invasion of cancer cells in triple-negative breast cancer and predicts patients’ survival by downregulating miR-21

**DOI:** 10.1186/s12885-026-16506-0

**Published:** 2026-07-20

**Authors:** Jianchao Gao, Sisi Wang, Zhisheng Zhang, Jun Li

**Affiliations:** 1 Department of Breast Surgery, The First Affiliated Hospital of Hebei North University, Zhangjiakou, Hebei Province 075000 People’s Republic of China; 2 Department of Obstetrics and Gynecology, The First Affiliated Hospital of Hebei North University, Zhangjiakou, Hebei Province 075000 People’s Republic of China; 3 Department of Radiotherapy, Jiangsu Cancer Hospital, Jiangsu Institute of Cancer Research, The Affiliated Cancer Hospital of Nanjing Medical University, Room 803, Building 19, Jinyu Zhongyang 2 Block, Shogunate West Road, Gulou District, Nanjing, Jiangsu Province 210009 People’s Republic of China

**BMC Cancer 21,745** **(2021)**

**https://doi.org/10.1186/s12885-021-08294-6**

The Editors have retracted this article. After publication, concerns were raised about some of the data in this article. Specifically, similarities have been detected between:

• Fig. 6A (BT-549/BRE-AS1) and Fig. 6B (BT-549/NC).

• Fig. 6A (BT-549/miR-21) and Fig. 6B (BT-549/miR-21).

• Fig. 6B (BT-549/BRE-AS1) and Fig. 6B (BT-549/BRE-AS1 + miR-21).

• Fig. 6A (HCC70/C) and Fig. 6A (HCC70/NC).

• Fig. 6A (HCC70/BREA-AS1) and Fig. 6B (HCC70/C).

The Authors have not responded to these concerns. Therefore, the Editors have lost confidence in the data and conclusions of this article.

Authors Jianchao Gao, Sisi Wang, Zhisheng Zhang, and Jun Li did not respond to correspondence from the Publisher about this retraction.

